# An interactive atlas of genomic, proteomic, and metabolomic biomarkers promotes the potential of proteins to predict complex diseases

**DOI:** 10.21203/rs.3.rs-3921099/v1

**Published:** 2024-03-05

**Authors:** Mikael Benson, Martin Smelik, Xinxiu Li, Joseph Loscalzo, Oleg Sysoev, Firoj Mahmud, Dina Mansour Aly, Yelin Zhao

**Affiliations:** Karolinska Institute; Karolinska Institute; Karolinska Institute; Brigham and Women’s Hospital; Linköping University; Karolinska Institute; Karolinska Institute; Karolinska Institute

## Abstract

Multiomics analyses have identified multiple potential biomarkers of the incidence and prevalence of complex diseases. However, it is not known which type of biomarker is optimal for clinical purposes. Here, we make a systematic comparison of 90 million genetic variants, 1,453 proteins, and 325 metabolites from 500,000 individuals with complex diseases from the UK Biobank. A machine learning pipeline consisting of data cleaning, data imputation, feature selection, and model training using cross-validation and comparison of the results on holdout test sets showed that proteins were most predictive, followed by metabolites, and genetic variants. Only five proteins per disease resulted in median (min-max) areas under the receiver operating characteristic curves for incidence of 0.79 (0.65–0.86) and 0.84 (0.70–0.91) for prevalence. In summary, our work suggests the potential of predicting complex diseases based on a limited number of proteins. We provide an interactive atlas (macd.shinyapps.io/ShinyApp/) to find genomic, proteomic, or metabolomic biomarkers for different complex diseases.

## Introduction

The shifting landscape of global healthcare towards complex diseases affecting the immune, metabolic, respiratory, and vascular systems has underscored the need for accurate biomarkers for early prediction or diagnosis. Such biomarkers are often prioritised based on the scientific literature, clinical experiences, or analyses of different omics data. This may be confounded by knowledge biases or, in the case of omics data, limited sample numbers.

Longitudinal studies have proved to be promising in searching for biomarkers. For example, analyses of proteomes and metabolomes in blood samples have shown associations with disease-associated traits^1^, as well as various immunological, cardiovascular or metabolic diseases^2–4^. UK Biobank (UKBB) is prospective study of some 500,000 individuals, which makes extensive phenotypic and multiomics data available to researchers across the globe. Since it has longitudinal data, it is possible to identify biomarkers for both patients that are already diagnosed and patients that will get diagnosed in the future (henceforth referred to as prevalent and incident disease, respectively)^5^. Analyses of genomics and proteomics data from UKBB and other resources have already resulted in multiple potential biomarkers^6–9^. These publications focused on applying proportional hazard models and included only incident cases. In this manuscript, we aimed to broaden the analysis by adding metabolomic data, as well as by using models that are applicable on both prevalent and incident cases.

While the abovementioned findings support the potential to identify biomarkers for complex diseases from multiomics data, an unanswered question is which type of molecule has the best potential to predict the disease. Another question is whether a single molecule or a limited number of molecules suffice to predict complex diseases that, in contrast to monogenic diseases, are caused by multiple interacting molecules^4,10^.

**Figure 1:**
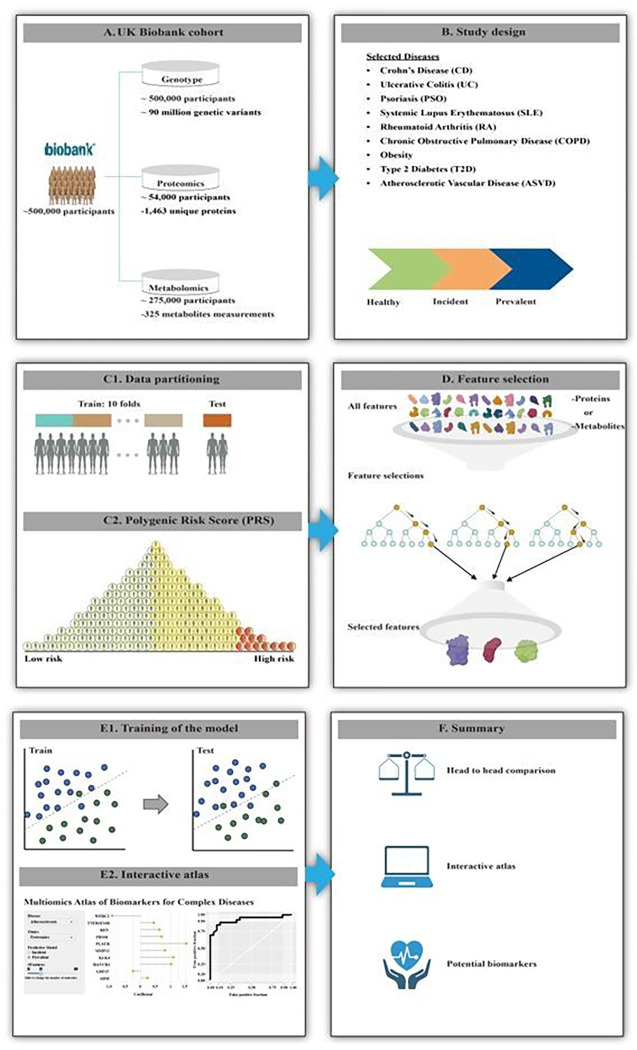
Overview of the study. **A)** Genomic, proteomic, and metabolomic data from patients with **B)** nine incident or prevalent complex diseases and age/sex matched controls were **C1)** analysed using cross-validation and holdout test datasets, and **C2)** A polygenic risk score was computed for genomic data, while **D)** feature selection was performed for proteomics and metabolomics. **E1)** A machine learning model was trained and tested to **E2)** construct an interactive atlas that can be found at macd.shinyapps.io/ShinyApp/. **F)** In summary, we present a comparison of different omics layers, and an interactive atlas to derive context-dependent types and numbers of potential biomarkers for incidence and prevalence of the diseases

This problem is illustrated by a recent study, which identified multiple disease-associated metabolites, each of which could vary greatly between both healthy and sick individuals^11^. To answer these questions, we performed a comparison of genomic, proteomic, and metabolomic data from the UKBB. We used machine learning to build predictive models for different combinations of genetic variants, proteins, and metabolites, and these models were utilized to search for potential biomarkers for the incidence and prevalence of nine complex diseases (Fig. 1).

## Results

### Patient cohort

Our analyses were based on 92,916 patients with rheumatoid arthritis (RA), systemic lupus erythematous (SLE), ulcerative colitis (UC), Crohn’s disease (CD), psoriasis (PSO), type 2 diabetes (T2D), obesity, atherosclerotic vascular disease (ASVD), and chronic obstructive pulmonary disease (COPD), as well as their age/sex matched controls. These diseases were selected because they had enough samples to perform statistically robust predictions. For each disease, we divided patients into those who were diagnosed after the assessment visit and those who were already diagnosed (incident and prevalent disease, respectively). The patient characteristics are presented in Supplementary Table 1.

### Interactive web-based atlas enables the search for biomarkers

The UKBB data that we analysed consists of genotypes data of 90 million genetic variants, 1,453 proteins, and 325 metabolite measurements. To find the optimal type and number of potential biomarkers for incidence and prevalence, we used a machine learning pipeline, which consisted of data cleaning, data imputation, feature selection, and model training, with comparisons of the results on holdout test sets. In all models, the data were divided into training and testing datasets. The classification model was trained using a 10-fold cross validation. The results are presented in an interactive web-based atlas (macd.shinyapps.io/ShinyApp/), which generates receiver operating characteristic (ROC) curves for the incidence or prevalence of each disease based on user-selected numbers of either proteins or metabolites. In the case of genomics, the ROC curves are generated from published polygenic risk scores (PRS)s for the studied diseases, which were derived from the polygenic score catalog^12^.

### Proteomics biomarkers outperforms biomarkers from other omics.

**Figure 2:**
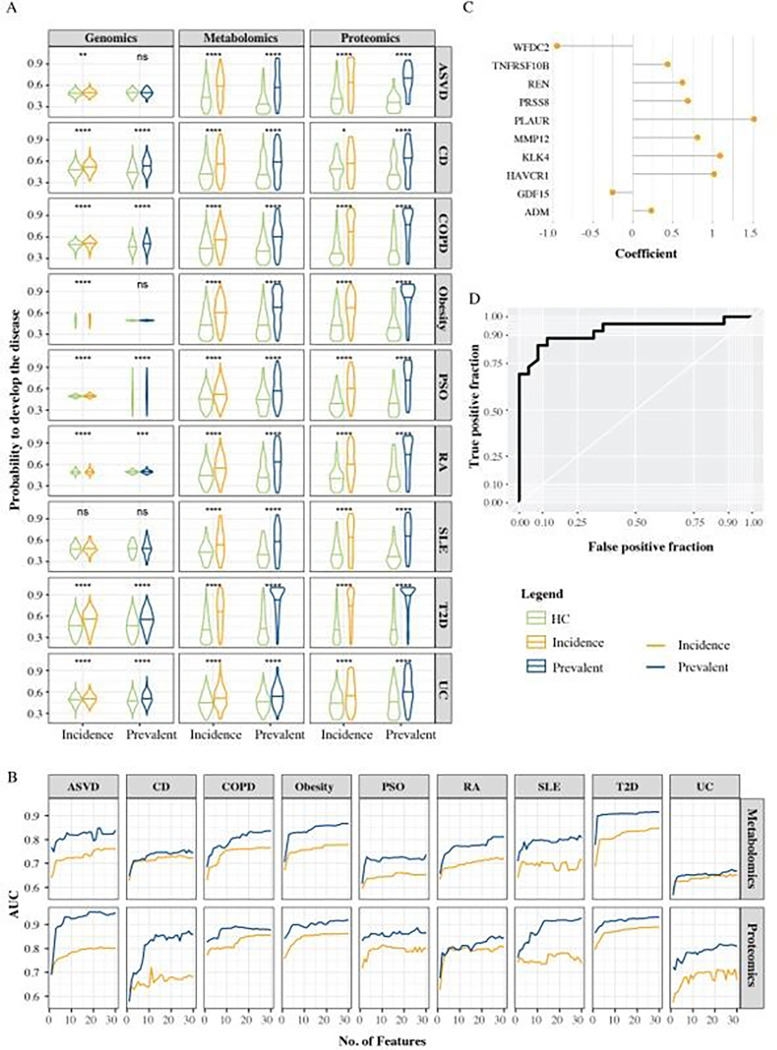
**A)** Violin plots showing the probabilities of incident and prevalent diseases, as well as healthy controls, based on PRS scores and five molecules derived from proteomic and metabolomic data. The centre line corresponds to median; **B)** line plots showing the relationship of the AUC of incident and prevalent diseases and the number of molecules used in the model; **C)**top 10 proteins for the ASVD prevalence, biomarkers with positive coefficients may have disease-inducing roles, while negative coefficient indicate protective roles; **D)** the ROC for ASVD prevalence. ASVD=Atherosclerotic Vascular Disease, CD=Crohn’s Disease, COPD=Chronic Obstructive Pulmonary Disease, PSO=psoriasis, RA=Rheumatoid Arthritis, SLE=Systemic Lupus Erythematosus, T2D=Type 2 Diabetes, UC=Ulcerative Colitis...* p<0.05, ** p <0.01, *** p < 0.001, **** p<0.0001. T test, adjusted p values

To evaluate the predictive performance of the different molecular types for incidence and prevalence of each disease, we started by computing test ROC curves based on only five proteins or metabolites, while for genomics, we used the individual scaled PRSs. We found that there was a significant difference between incidence or prevalence and healthy controls for all diseases in proteins and metabolites, while the differences in PRSs were sometimes not significant (Fig. 2A). The boxplots for proteins and metabolites also indicate that healthy controls can be separated from incident and prevalent cases with a good precision. To further evaluate the models, we computed the area under the ROC curves (AUCs). We aimed for AUCs of 0.8 or greater, which may be of clinical significance^13^. Overall, proteins yielded the highest AUCs. The AUCs reached 0.8 or more for all diseases except CD and UC. The median (min-max) AUC for incidence was 0.79 (0.65–0.86), and for prevalence 0.84 (0.7–0.91). Metabolites yielded median (min-max) AUCs for incidence and prevalence of 0.70 (0.62–0.80) and 0.86 (0.65–0.90), respectively. T2D, obesity and ASVD had the highest AUCs. Genetic variants resulted in median AUCs for incidence and prevalence of 0.57 (0.53–0.67) and 0.6 (0.49–0.70), respectively. The most clinically significant AUCs were found in CD, PSO and T2D. These findings suggested that as few as five proteins may suffice for both predicting incident and diagnosing prevalent disease. However, the optimal number could be context dependent. For example, a serious disease may motivate a larger number of biomarkers than a less serious one. To address this question, we analysed the atlas to compute AUCs for different numbers of proteins and metabolites (Fig. 2B,C,D and Supplementary Table 2). For most of the diseases, five or fewer proteins sufficed to achieve AUCs of 0.8 or more. For example, in ASVD only three proteins resulted in an AUC of 0.88 for prevalence, namely, matrix metalloproteinase 12 (MMP12), TNF Receptor Superfamily Member 10b (TNFRSF10B), and Hepatitis A Virus Cellular Receptor 1 (HAVCR1), consistent with extant knowledge on the role of inflammation and matrix degradation in atherogenesis. However, for incidence, 18 proteins were needed to achieve an AUC of 0.8 (Fig. 2B).

## Discussion

Our comparison of genomic, proteomic, and metabolomic data provides a systematic solution for the prioritisation of the type and number of potential biomarkers for the incidence and prevalence of nine common complex diseases. The clinical relevance lies in that prioritisation of biomarkers is complicated by each disease involving thousands of genes and gene products that can vary between the same patient before and after diagnosis, as well as between patients with the same diagnosis. Thus, biomarker prioritisation based on literature, clinical experience, or omics data involves formidable challenges. The main finding of our study is that a limited number of proteins have potential for both prediction and diagnosis, representing substantial dimensionality reduction of the ever-expanding pool of big data acquired from patients with these complex disorders.

From a clinical perspective, an advantage of proteins is that they can be measured with routine clinical methods. For any biomarker and disease, the optimal number of proteins is a trade-off between cost, sensitivity and specificity^13,14^. For example, the prediction or diagnosis of a serious disease may motivate a larger number of proteins than a less serious disease. We make all the molecule combinations and their AUCs available to facilitate systematic and context-dependent prioritisation of biomarkers for clinical studies.

It may be difficult to discern whether different omics layers have casual effect in relation to the disease mechanisms or rather reflect the consequences of those mechanisms. For example, we have previously found that different subtypes of diabetes have variable genetic and environmental associations^15^. This heterogeneity is particularly evident in type 2 diabetes, which has a strong genetic component. However, environmental factors, like diet also play a large role. In agreement with this, our analyses of type 2 diabetes, showed that metabolites and proteins had higher AUCs than genetic variants. While the genetic variants likely have causal roles, it is difficult to define if the metabolites or proteins change because of the environmental factors or secondary to intrinsic disease mechanisms. Further studies are warranted to offer a better understanding of the role of biomarkers in disease progression. Because of its longitudinal design, the UKBB provides a unique opportunity to identify incidence biomarkers that are potentially associated with early disease mechanisms. Proteins in blood may be particularly suitable because they reflect changes in tissues and mediate a wide range of disease-relevant functions, such as interactions between cells, immune responses, vascular functions, tissue remodelling^16,17^. Thus, the incidence biomarkers may help to discover, or prioritise among previously known, early disease mechanisms and thereby identify targets for preventive treatment.

As one example, ASVD is an important cause of morbidity and mortality worldwide and is associated with myocardial infarction, stroke, vascular dementia, and peripheral arterial occlusive disease. Early prediction of subclinical disease and prevention or treatment are, therefore, key health care objectives with the potential to greatly reduce patient suffering^18^. The incidence biomarker proteins identified by our analysis are mechanistically rational (although they need not be a priori) and are responsible for inflammatory responses in the early plaque (CXCL17, PLAUR), stress responses after (hypoxic or inflammatory) injury (GDF15), innate immunity (WFDC2), and angiogenesis (PLAUR, WFDC2). Similarly, as discussed above, the prevalent biomarker proteins include proteins critical for matrix remodelling (MMP12, KLK4), cytokine-mediated inflammatory responses (TNFRSF10B), and Hif-1alpha-dependent angiogenesis (ADM).

Limitations of our study include the fact that proteins and metabolites in blood may not reflect disease-associated changes in tissues or may vary for reasons other than disease. Moreover, a limited number of proteins and metabolites were analysed, with technologies that could have method-dependent variations. Variable numbers of patients and controls were used for different omics layers, and thus, the statistical power of proteomics is lower than the other layers. Another limitation is that the UKBB mainly consists of European participants with restricted age ranges. These limitations point to how our atlas can be exploited to facilitate future studies of 1) more diverse populations, starting with targeted analyses of prioritised biomarkers rather than with more costly omics technologies; 2) combinations of prioritised biomarkers and clinical variables. The relevance lies in the fact that clinicians usually make diagnostic decisions based on combining biomarkers with routine clinical variables; 3) investigation of data integration methods to construct classifiers with the capacity to incorporate different omics layers; and 4) incidence biomarkers to predict and potentially prevent complex diseases. The importance lies in the fact that many complex diseases have vague or no symptoms at early stages but are easier to treat at that point rather than at later stages. A well-known example is how biomarkers for early diagnosis have greatly improved the treatment of RA. However, finding targets for early treatment is complicated by systematic studies of early mechanisms being difficult to perform in human subjects before diagnosis. In summary, we found that a limited number of proteins in blood may suffice for the early prediction and diagnosis of complex diseases. We make those proteins, as well as metabolites and genetic variants associated with the PRS of the diseases, available in the form of an interactive atlas for future studies to evaluate their potential.

## Methods

### Data source and participants

Participants of this study were a part of the UKBB dataset, a large prospective cohort study consisting of more than 500,000 participants recruited in the United Kingdom^5^. Full details of the UKBB study can be found on the UKBB website (https://biobank.ndph.ox.ac.uk/showcase/). UKBB received ethical approval from the National Information Governance Board for Health and Social Care and the National Health Service Northwest Multi-Center Research Ethics Committee^5^. All participants gave informed consent through electronic signatures before enrolment in the study. This research has been conducted under approved UKB Project ID 102162. The follow up of the individuals was until the 31st of October 2022.

The specific data fields used in this analysis were nuclear magnetic resonance (NMR) metabolomics, proteomics, imputed genomic data, date of recruitment, age, sex, date of diagnosis, and Diagnostic Codes-ICD10.

All methods were performed in accordance with the relevant guidelines and regulations or declaration of Helsinki.

### Data processing

NMR spectroscopy measurements took place between June 2019 and April 2020 (Phase 1), April 2020 and June 2022 (Phase 2), using eight spectrometers at Nightingale Health based in Finland. The metabolic biomarkers are involved in multiple metabolic pathways, including lipoprotein lipids in 14 subclasses, fatty acids, and fatty acid composition, as well as various low-molecular-weight metabolites, such as amino acids, ketone bodies, and glycolysis metabolites quantified in molar concentration units. The dataset comprised 249 NMR metabolite measurements along with their associated quality control (QC) matrices. Of these measurements, 168 were absolute, and 81 were ratios. Data preprocessing, technical variation removal, and computation of an additional 76 biomarkers from the post-QC dataset were conducted using the ukbnmr package (version 2.0)^19^. Consequently, 325 metabolite measurements were utilised in subsequent analyses. For individuals with both repeat assessments (2012–2013) and baseline assessments (2006–2010), only the baseline data were retained. Repeated measures were based on the Eid and visit_index columns from the UKBB dataset. Technical variations were removed using the updated Algorithm version 2 in the ukbnmr V2 package, where well positions within each batch were separately considered and adjusted. Further details on this approach can be found at the ukbnmr GitHub repository.

Proteomic profiling of blood plasma samples was collected during participant visits between 2006 and 2010 (UKBB dataset field: 53) using the Olink Explore 1536 platform, measuring 1,472 protein analytes and capturing 1,463 unique proteins. The criteria for participant inclusion in the UK Biobank Pharma Proteomics Project (UKB-PPP) and the specifics of the proteomics assays and normalisation processes are detailed in an earlier study^20^.

Genetic data was downloaded from UKBB. The genotyping and imputation (and quality control) were performed by the UKBB^21^. Genome-wide data available from the UK Biobank v3 imputed data in BGEN v1.2 format.

The NMR measurement, proteomic profiling, and genomic data were processed using code 3, 143, and code 87, which enabled the decoding of the data.

### Patient stratification

The 10th revision of International Classification of Diseases (ICD10) codes was used to assess the diagnosis of the patients. We identified patient groups based on ICD10 codes in the hospital inpatient data (UKBB datasets field: 41270), which is curated from UKBB as provided. In the analysis, we included Crohn’s disease (CD) (K50), ulcerative colitis (UC) (K51), psoriasis (L40), systemic lupus erythematosus (SLE) (M32), chronic obstructive pulmonary disease (COPD) (J449), obesity (E66), type 2 diabetes (T2D) (E11), atherosclerotic vascular disease (ASVD) (I70), and rheumatoid arthritis (RA) (M05, M06). In case of genomics, we only selected patients with European origin. We distinguished incident and prevalent cases based on the earliest reported data across the respective date of first inpatient diagnosis (fields 41262 and 41280) columns from the UKBB. Thus, individuals receiving a diagnosis after the time of sampling were labelled incident cases, and patients diagnosed before or at the time of sampling were classified as prevalent cases. For each group of patients, we identified a group of healthy controls, which were defined as all patients without any disease code. We used the MatchIt package^22^ to match the healthy control cases with the prevalent and incident cases based on age and sex. The match was performed by the nearest neighbour method using Euclidean distance as a measure of similarity. We used exact matching for sex.

### Weighted PRS analyses.

The known PRS from the polygenic score catalog for each disease was used to determine the genetic contribution to the probability of developing the disease^12^. The known PRS and genetic variants for each disease can be found in Supplementary Table 1. IMIDs were RA (PGS000194), SLE (PGS000328), UC (PGS001306), CD (PGS001331), and PSO (PGS002293). Chronic diseases included COPD (PGS001332), T2D (PGS000864), obesity (PGS000848), and ASVD (PGS000863). Imputed genetic data (930995623 genetic variants) from UKBB were used to calculate the corresponding PRSs (1595 genetic variants in PRSs) for everyone in each disease separately. Genetic variants for each PRS were pruned for linkage disequilibrium (r2 = 0.5, 250-kb window in PLINK). The weighted PRSs were calculated for all participants included in the analysis of each disease using the standard formula in PLINK v1.9 software^23^. All analyses of the PRSs included participants of European origin only and were adjusted for the first 3 genetic principal components supplied by the UKBB quality control files^15^. The principal components were generated by UKBB^21^. The list of SNPs for each disease is available in supplementary table S3.

### Modelling the probability of health status

To construct the classifiers, we created a pipeline consisting of data cleaning, data imputation, feature selection, training of the classifier and summary of results. In detail, we first removed all molecules with more than 10% NA values and divided the data into training (70%) and testing (30%) groups. We then trained a KNNImputer^24^ method to impute NA values. The choice of the KNN method was based on the computational efficiency, simplicity and a recent study^25^, which suggested that KNN has a comparable performance to other more complex methods for the continuous data, in our case proteomics and metabolomics, with a low amount of missing values. Next, we applied the extremely randomised trees (ERT)^26^ method for feature selection. We used 10000 trees, and the main motivation for using ERT was the reduced impact of multicollinearity, which is handled by using multiple trees and utilising random splits. The feature selection ranked molecules from the most important to least important. For the downstream analysis, we chose only the N most important features, where N (1–30) was selected by the user. To train the prediction model, we used a logistic regression with a ridge (L2) penalty^27^, with the N most important proteins as features and binary disease status as the response variable. Even though it may slightly reduce the performance of the model, the use of logistic regression with the L2 penalty over more complex nonlinear methods is motivated by the interpretability of the method, which we consider essential in clinical research. Furthermore, logistic regression is a tool with which many clinicians are familiar and provides us with the possibility to assess whether the feature has a positive or negative association with the disease. The disease status was dependent on the user input. In the case of ‘incident’, the aim of the model was to discriminate the incident cases from healthy controls matched by age and sex. Analogously, when setting ‘prevalent’, the aim was to discriminate prevalent cases from their respective healthy controls. We used cross-validation as implemented in the LogisticRegressionCV^28^ method from the sklearn package to optimise the L2 penalty factor. All the above was performed per training dataset. To measure the performance of the whole pipeline, we applied the trained imputation model, downsampled the feature set based on the trained ERT model and applied the trained logistic model to the test datasets and presented the receiving operating characteristic (ROC) curve together with its corresponding area under the ROC curve (AUC). The same pipeline was used for proteomics and metabolomics. For PRSs, we used the same pipeline in which the feature selection step was omitted. Instead, we used sex, age, scaled PRS and three genetic principal components (these are generated from the QC standard pipeline of the genotyping data^29^ and are supplied by the UKBB for all individuals)^15^ as features and patient group as response variable. We chose these features because they are commonly used in the scientific literature. We used the ggpubr^30^ package to perform a two-sided t test with a false discovery rate adjustment to determine whether the probability of developing disease was significantly different between healthy controls and incident or prevalent cases. More specifically, we tested whether the mean of the test predictions from the logistic regression for incident or prevalent cases differed significantly from the mean of the test predictions from the logistic regression for healthy controls. The analysis was performed using R (v4.1.1) and Python (v3.7.9), and we used default parameters and random seed 42 for all analyses unless otherwise stated.

### Construction of the multiomics atlas

To simplify the interpretation of the results, we created a shiny app^31^. The app can be found at macd.shinyapps.io/ShinyApp/. All the results shown in the atlas are based on the predictive models explained above. For each disease, the atlas provides context-dependent options for adjustments, namely, the type of 1) predictive model, 2) omics layer, and 3) number of molecules. The atlas predicts a set of biomarkers based on those settings. There are three omics layers, namely, genomics, proteomics, and metabolomics, from which a user can choose. To assess the discriminative performance, a receiver operating characteristic (ROC) curve for the test dataset is presented. If the predictive model is set to “incident”, the atlas generates AUCs for incident cases versus healthy controls for different types and numbers of molecules. Analogously, the setting “prevalent” generates a test set for prevalent cases. We provide a choice of one to thirty biomarkers.

## Data Availability

An interactive, web-based atlas for translational researchers to find optimal biomarkers is available at macd.shinyapps.io/ShinyApp/. All data used in this study are available to access from the UK Biobank at https://www.ukbiobank.ac.uk/ for approved researchers through the UK Biobank data-access protocol.
